# Do Opposites Attract? Auxin-Abscisic Acid Crosstalk: New Perspectives

**DOI:** 10.3390/ijms24043090

**Published:** 2023-02-04

**Authors:** Paloma Ortiz-García, Adrián González Ortega-Villaizán, Francis Chukwuma Onejeme, Maren Müller, Stephan Pollmann

**Affiliations:** 1 Centro de Biotecnología y Genómica de Plantas, Instituto Nacional de Investigación y Tecnología Agraria y Alimentación (INIA/CSIC), Universidad Politécnica de Madrid (UPM), Campus de Montegancedo, Pozuelo de Alarcón, 28223 Madrid, Spain; 2 Department of Evolutionary Biology, Ecology and Environmental Sciences, Faculty of Biology, University of Barcelona, 08028 Barcelona, Spain; 3 Departamento de Biotecnología-Biología Vegetal, Escuela Técnica Superior de Ingeniería Agronómica, Alimentaria y de Biosistemas, Universidad Politécnica de Madrid (UPM), 28040 Madrid, Spain

**Keywords:** indole-3-acetic acid, abscisic acid, plant hormones, crosstalk, signaling

## Abstract

Plants are constantly exposed to a variety of different environmental stresses, including drought, salinity, and elevated temperatures. These stress cues are assumed to intensify in the future driven by the global climate change scenario which we are currently experiencing. These stressors have largely detrimental effects on plant growth and development and, therefore, put global food security in jeopardy. For this reason, it is necessary to expand our understanding of the underlying mechanisms by which plants respond to abiotic stresses. Especially boosting our insight into the ways by which plants balance their growth and their defense programs appear to be of paramount importance, as this may lead to novel perspectives that can pave the way to increase agricultural productivity in a sustainable manner. In this review, our aim was to present a detailed overview of different facets of the crosstalk between the antagonistic plant hormones abscisic acid (ABA) and auxin, two phytohormones that are the main drivers of plant stress responses, on the one hand, and plant growth, on the other.

## 1. Introduction

Global warming and environmental pollution are among the major threats that humanity will face in the coming decades. Rising global temperatures and more frequent heat waves accompanied by unforeseeable torrential rains [1,2] are assumed to substantially exacerbate the conditions of abiotic stress in which plants will have to survive and prosper in the future. These unfavorable conditions are expected to have detrimental effects on crop quality and productivity [3]. Abiotic stress cues affect plant morphology, biochemistry, physiology, and anatomy by altering biological processes as diverse as photosynthesis, respiration, growth, and development. Moreover, long-term stress can finally lead to plant death [4]. Plants have developed sophisticated gene regulatory networks and defense mechanisms to face abiotic stresses [5,6]. Drought and increased soil salinity affect plant yields [7,8], and if we add the increasing population that humanity is experiencing, the problem is exacerbated. By 2050, the world population is expected to reach around nine billion [9,10] and, consequently, the availability of arable land will decrease [9]. The global demand for crops will grow 100–110% [11] and one solution we have is to improve plant yields to secure future food supply by developing genetic strategies that allow crops to tolerate abiotic stresses caused by climate change [12].

Plants perceive changes in their environment and, in turn, modulate the content and distribution of a small number of metabolites, referred to as plant hormones, which serve the integration and transmission of internal signals [13,14,15]. ‘Hormone’, from Greek hormān, meaning ‘to stimulate’ or ‘to set in motion’ in English, refers to small bioactive signaling molecules that can exert their action at a very low, submicromolar concentration [16]. Throughout evolution, plants have developed a set of these small molecules that play key roles in the regulation of plant growth and development, as well as in the development of adequate responses to biotic and abiotic stress cues [17,18]. Currently, nine different classes of plant hormones are considered and classified according to their major effect on plant growth. The common classification includes, on the one hand, stress response-related hormones, i.e., abscisic acid (ABA), ethylene, salicylates (SAs), and jasmonates (JAs), and, on the other hand, growth-promoting hormones, including the classes of auxins, gibberellins (GAs), cytokinins (CKs), brassinosteroids (BRs) and strigolactones (SLs) [19,20,21]. For a hormone to be capable of triggering its physiological function, it must first be recognized by its receptor(s); subsequently, its signaling pathway must be activated, which will ultimately result in transcriptional modifications driven by the activation or inactivation of a given number of specific plant hormone-responsive transcription factors [15].

However, many recent studies indicated that the classification of plant hormones, according to their growth-promoting or -repressing effects, is all too simplistic and needs to be readdressed because many interactions between antagonistic plant hormones have been described in the past. Just to name some examples, wound induced formation of JA has been shown to trigger auxin biosynthesis in *Arabidopsis thaliana* through the induction of *YUCCA8* and *YUCCA9* gene expression, involving the transcriptional control of target genes by transcription factors from the JA-specific basic helix loop helix family myelocytomatosis oncogene (MYC) [22,23]. Moreover, auxin and JA have been described to collaborate in flower development [24,25], and the signaling pathways of both hormones are known to share some common components, including the Arabidopsis S-PHASE KINASE-ASSOCIATED PROTEIN 1 (ASK1) and TOPLESS (TPL) [17] while auxin and ethylene are interacting in controlling fruit ripening in fruits [26,27]. This review provides updated information on the molecular intricacies by which the main plant growth factor, indole-3-acetic acid (IAA), interacts with the growth repressing phytohormone ABA, thus contributing to orchestrate plant growth under abiotic stress conditions that can arise from the global climate change scenario. The discussed novel insight into the crosstalk between IAA and ABA is assumed to open new possibilities for biotechnological innovations that will lead to sustainable solutions to improve agricultural productivity and help ensure food security in the long run.

## 2. Auxins

The class of auxins contains the most important phytohormones related to plant growth and development. Auxins are involved in processes ranging from cell expansion growth, control of cell division, vascular tissue differentiation, lateral and adventitious root initiation, tropistic responses (gravitropism and phototropism), maintenance of apical dominance, delay of leaf senescence, and contributions to the control of leaf and fruit abscission, as well as fruit ripening [28,29].

### 2.1. Auxin Biosynthesis

IAA is considered the most abundant and most important representative of the auxin class in the plant kingdom. Its occurrence in plants was first described nearly a century ago by Frits W. Went and Kenneth V. Thimann [30,31], but despite decades of intense efforts, the biosynthesis of this first identified plant hormone remained enigmatic until a few years ago. For a long time, two different biosynthetic starting points were discussed in the literature. For one thing, auxin biosynthesis pathways dependent on l-tryptophan (l-Trp), including the indole-3-pyruvic acid (IPyA) pathway [32,33,34,35,36], the main auxin biosynthesis pathway in plants and the only biosynthetic route fully disclosed to date leading to auxin in higher plants, were suggested. On the contrary, an l-Trp-independent auxin biosynthesis pathway was introduced more than 30 years ago [37,38], although clear genetic evidence was lacking and mass spectrometric findings were controversially discussed in the literature [39]. However, a more recent study describing the presumably crucial role of the indole synthase complex during embryogenesis revived the discussion of an l-Trp-independent auxin biosynthesis pathway [40].

Regarding the auxin biosynthesis pathways dependent on l-Trp, together with the IPyA pathway for IAA biosynthesis that involves the conversion of l-Trp to IPyA by means of two l-Trp aminotransferases, TRYPTOPHAN AMINOTRANSFERASE OF ARABIDOPSIS 1 (TAA1) and TRYPTOPHAN AMINOTRANSFERASE-RELATED 2 (TAR2) [33,41], and the subsequent enzymatic transformation of IPyA into IAA by flavin-containing monooxygenases of the YUCCA family [32,35,42], three additional pathways have been described: the tryptamine (TAM)-, the indole-3-acetamide (IAM)-, and the indole-3-acetaldoxime (IAOx) pathway [43,44,45]. While the TAM pathway does not appear to be of great relevance for auxin de novo biosynthesis, as there is no evidence that the inactivation of the involved aldehyde oxidases in the *aba3* mutant translated into altered IAA levels in Arabidopsis [35,36], it must be concluded that the two other pathways may play minor or specialized roles in auxin biosynthesis. The occurrence of IAOx has initially been limited to members of species of the Brassicaceae family, as outside of the Brassicas, no IAOx producing cytochrome P450 monooxygenases (CYP79B2, CYP79B3) have been described [46,47]. Recent publications challenged this notion, reporting either the identification of CYP79 enzymes capable of producing IAOx or the conversion of IAOx into IAA in non-Brassica species, including, for instance, poplar, maize, and *Medicago truncatula* [48,49,50]. Interestingly, it is suggested that IAOx is the main precursor of IAM in Arabidopsis [51], but the enzyme that converts IAOx to IAM remains elusive. In addition to that, the main role of the IAOx pathway is probably the production of secondary plant metabolites, including indole glucosinolates, camalexin, and indole-3-carboxylic acid, which are crucial for defense reactions in Arabidopsis [52]. The IAM pathway has long been thought to be restricted to bacteria that use this two-step pathway involving a tryptophan 2-monooxygenase (iaaM/tms1/aux1) and an IAM hydrolase (iaaH/tms2/aux2) to convert l-Trp into IAA [53]. Nonetheless, nearly 20 years ago, the first plant IAM hydrolase gene from Arabidopsis, *AMIDASE 1* (*AMI1*), was cloned and several other AMI1-like enzymes have been characterized from a multitude of other plant species since then [54,55,56]. AMI1 belongs to the amidase signature (AS) family of amidohydrolases, of which at least one other member, FATTY ACID AMIDE HYDROLASE (FAAH), is involved in terminating the signaling activity of other signaling molecules, *N*-acylethanolamines [57,58]. Along with AMI1, a recently described genetic screen suggested the involvement of two additional putative IAM hydrolases, IAMH1 and IAMH2, in the conversion of IAM, but so far no reports on their enzymatic characterization have been provided, and the double mutant does not show significant differences in its IAA content under control conditions but only when IAM is applied exogenously, suggesting a possible unspecific activity of IAMH1 and IAMH2 when IAM levels are artificially increased [59]. The latter observation stands in clear contrast to the recently reported findings for two *ami1* alleles that both showed significantly reduced IAA levels and elevated IAM contents under control conditions [60]. Along with the broad distribution of AMI1-like enzymes in the plant kingdom, this implies a more specific role of AMI1-like IAM hydrolases in plants.

### 2.2. Auxin Transport

For proper plant growth and development, strict control of cellular auxin homeostasis and auxin fluxes along the plant body is imperative. Auxin gradients produced in plant tissues change dynamically due to developmental programs and in response to external stimuli [61,62]. In this context, auxin transport plays a key role. In general, plants possess two different auxin transport systems. On the one hand, plants transport auxin non-directionally in the phloem to recipient organs, together with the photo-assimilates. On the other hand, they possess directional polar auxin transport (PAT), which distributes auxin in a spatially precise manner. The latter transport system is the main contributor to the generation of auxin gradients across plant tissues [63] and fundamental for generating local auxin maxima [64]. The formation of these maxima is of paramount importance for many developmental processes, such as phyllotaxis, as well as flower and lateral root development [65,66,67], or to generate adequate responses to external stimuli, including phototropism [68].

The acidic pH of the apoplast (pH~5.5) [69], in contrast to the neutral pH (pH~7) of the cytoplasm, allows passive diffusion of auxin across cell membranes, although auxin can also enter the cell via specialized transporters of the AUXIN RESISTENT 1/LIKE AUX1 family (AUX1/LAX) [70]. Due to the low pH in the apoplast, IAA occurs in its protonated state (IAAH), while in the cytoplasm IAA dissociates into its ionic form (IAA^−^), preventing the passive efflux of the molecule from the cell [64]. Auxin efflux depends on auxin transporters of the PIN-formed (PIN) family or P-glycoprotein/ATP-binding cassette B (ABCB) transporters. PIN1-4 and PIN7 are located in the plasma membrane, where they mainly contribute to auxin efflux to the apoplast. In contrast, PIN5 and PIN8 are found in the endoplasmic reticulum together with PIN6, which can be found in both the plasma membrane and the endoplasmic reticulum [71,72]. However, the most remarkable feature of the PINs is their polar distribution in the plasma membrane, which allows for the directional flux of auxin and the formation of auxin maxima.

### 2.3. Auxin Signaling

**Figure 1:**
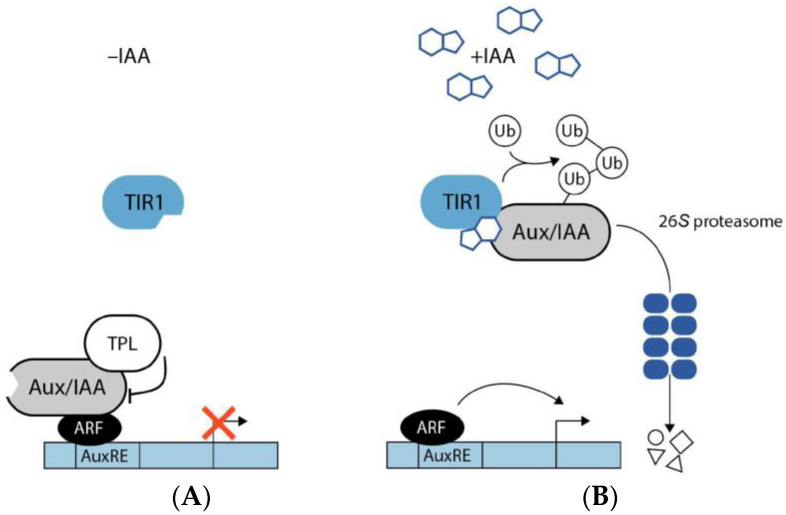
Auxin signaling pathway. (**A**) In the absence of IAA, the F-box protein TRANSPORT INHIBITOR RESPONSE 1 (TIR1), which acts as an auxin receptor, comprises no bound IAA molecules. Auxin/indole-3-acetic acid (Aux/IAA) proteins together with the co-repressor TOPLESS (TPL) interact physically with auxin response factor (ARF) transcription factors and, thereby, prevent the transcription of genes that contain auxin responsive elements (AuxRE) in their promoter sequence. (**B**) In the presence of IAA, the bioactive hormone binds to the binding cavity of the TIR1 receptor. Through the binding of IAA, TIR1 affinity for the Aux/IAA transcriptional repressors increases. Subsequently, Aux/IAA proteins are tagged with ubiquitin (Ub) for their degradation via the 26S proteasome. The ubiquitin-mediated proteolysis of Aux/IAA results in the liberation of the co-repressor TPL and the ARFs, thus, allowing ARFs to promote transcription of AuxRE containing genes.

Auxin signaling occurs both in the nucleus and in the plasma membrane. The core element that controls the signaling pathway is the E3 ubiquitin ligase complex SCF^TIR1/AFB^ that either contains the F-box protein TRANSPORT INHIBITOR RESPONSE 1 (TIR1) or a TIR1 homolog auxin-related F-box protein (AFB) [73], the co-receptor/repressor proteins auxin/indole-3-acetic acid (Aux/IAA) [74], and the auxin response factor (ARF) transcription factor family [75]. The receptor switches between two different activity states depending on the presence or absence of auxin (Figure 1).

In the absence of auxin, Aux/IAA repressor proteins heterooligomerize with the ARFs and the co-repressor TOPLESS (TPL), thereby inhibiting the transcriptional activity of the ARF transcription factors. However, in the presence of auxin, which binds at the bottom of an Aux/IAA protein-specific pocket of the F-box protein, the E3 ubiquitin ligase complex SCF^TIR1/AFB^ can recruit the Aux/IAA co-receptor proteins. Following binding, the Aux/IAA proteins are polyubiquitinated and thereby tagged for their subsequent proteolytic degradation by the 26S proteasome. The ARFs, in turn, are liberated from their repression and are free to mediate auxin-responsive transcriptional responses [76,77]. However, some very rapid auxin-triggered effects, including plasma membrane hyperpolarization and protoplast swelling that manifest within minutes or even seconds could not be attributed to the described gene regulatory system. Thus, for almost 50 years, the existence of a second auxin receptor responsible for fast responses has been hypothesized [78], but it was only very recently that the role of the long discussed best candidate, the plasma membrane localized AUXIN BINDING PROTEIN 1 (ABP1), in conferring rapid responses to auxin, was demonstrated beyond doubt [79].

## 3. Abscisic Acid

ABA is a sesquiterpene isoprenoid (C_15_H_20_O_4_). ABA biosynthesis commences in plastids, while the final steps occur in the cytosol [80]. It is obtained mainly from the cleavage of *β*-carotene (C40) [80,81,82], a derivative of the 2-*C*-methyl-d-erytritol (MEP) pathway, through multistep enzymatic catalysis. ABA received its name in 1968 [83], after being previously discovered and named as ‘inhibitor β’ and ‘dormin’, respectively [84], in studies on the control of leaf abscission and bud dormancy. Up to date, the role of ABA as a hormone involved in developmental processes is still being revisited. For example, basal ABA levels seem to play an indispensable role in xylem and leaf development [85], and promote crucial subcellular processes, such as chloroplast biogenesis [86]. For a more thorough review on ABA discovery, refer to [87].

### 3.1. ABA Biosynthesis

The formation of ABA involves a complex multi-step enzymatic conversion of *β*-carotene [88,89]. The first committed step in ABA biosynthesis occurs in plastids. Zeaxanthin or antheraxanthin is converted to all-*trans*-violaxanthin by the enzyme zeaxanthin epoxidase (ZEP), which is known as ABA1 in Arabidopsis, the only *ZEP* gene in this species. However, it should be noted that *aba1* mutants contain some residual ABA, suggesting that there is an alternative minor biosynthesis pathway operative in Arabidopsis [90]. Then, all-*trans*-violaxanthin is converted into 9-*cis*-violaxanthin or (via *trans*-neoxanthin) into 9′-*cis*-neoxanthin. In Arabidopsis, ABA4 produces *cis*-neoxanthin and *cis*-violaxanthin [91,92]. Subsequently, the ABA4 product is converted further to xanthoxin (C_15_) by 9-*cis*-epoxy carotenoid dioxygenases (NCEDs) by cleavage of 9-*cis*-violaxanthin or 9′-*cis*-neoxanthin. The NCED enzyme family has been found to require iron and oxygen to become catalytically active [93]. In Arabidopsis, NCED3 is thought to play a very prominent role in catalyzing the rate-limiting step in the biosynthesis of ABA [94]. Interestingly, *NCED3* gene expression has recently been associated with IAM abundance in Arabidopsis [59]. Xanthoxin is then transported to the cytosol, where the remaining reaction steps of ABA synthesis take place. In a first step, a short-chain alcohol dehydrogenase/reductase, ABA2 in Arabidopsis, converts xanthoxin to ABA-aldehyde [95], before ABA-aldehyde is finally oxidized to ABA by abscisic acid oxidases (AAOs). In Arabidopsis, ABSCISIC ALDEHYDE OXIDASE 3 (AAO3) is described to be the only AAO enzyme involved in ABA biosynthesis [96]. To exert its enzyme activity, AAO3 requires a sulfurated molybdenum cofactor (MoCo-S), which is provided by ABA3 in Arabidopsis [97] and FLACCA in tomato [98], respectively. Mutants in *ABA3* or *FLACCA* present an ABA-deficient phenotype [99,100].

### 3.2. ABA Transport

Classically, ABA was considered to show a root-to-shoot transport pattern, especially under drought stress. However, more recent studies have put this notion into question, and it is accepted today that ABA biosynthesis also takes place in aerial vascular tissues [101]. Several ABA transporters have recently been discovered, supporting the idea that ABA is transported throughout the plant by its specific transporters [102]. In this regard, proteins belonging to the P-glycoprotein/ATP-binding cassette G (ABCG) family have been described [103], both as exporters, as for instance ABCG25 [104], and importers, such as ABCG40 [105]. Moreover, members of this family participate in shoot-to-root ABA transport [106]. Other families which have been attributed to have an ABA transport activity include the nitrate transporters, e.g., ABA-IMPORTING TRANSPORTER 1/NITRATE TRANSPORTER 1.2(AIT1/NRT1.2) [107] and detoxification efflux carriers (DTX)/multidrug and toxic compound extrusion (MATE), such as DTX50 [108]. The observation that the NRT1/PTR family is involved in the transport of nitrogen-containing compounds, as well as ABA, is of special interest, since this opens new opportunities to study the intricacies of the relationship between shared ABA and nitrogen transport activities [109].

### 3.3. ABA Signaling

**Figure 2:**
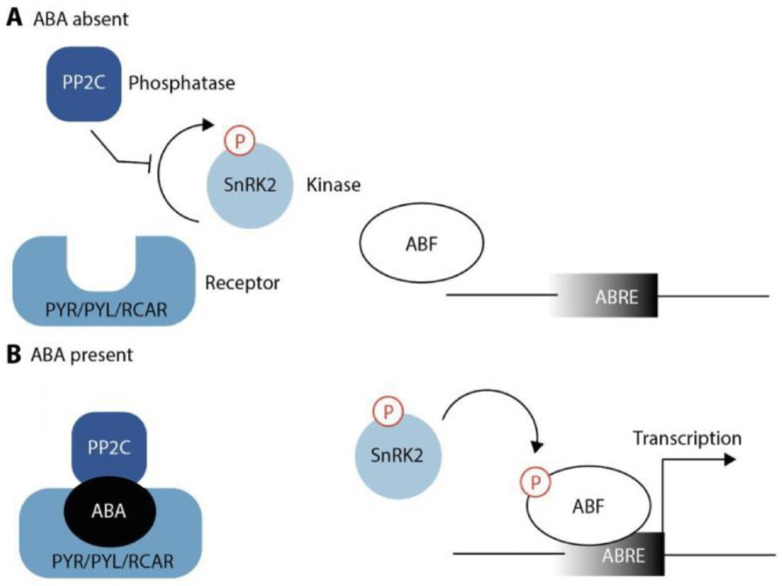
Schematic model of ABA signaling. (**A**) In the absence of ABA, the PP2C phosphatase is free to inhibit autophosphorylation of a family of SnRK kinases. (**B**) ABA presence enables the PYR/PYL/RCAR family of proteins to bind to and sequester PP2C. This releases the SnRK kinases from inhibition, which then become auto-activated and, in turn, obtain the capacity to phosphorylate and activate downstream ABA-responsive transcription factors (ABF) to initiate transcription of genes that contain ABA-responsive promoter elements (ABREs) in their regulatory sequences.

ABA perception and signaling are complex and involve the interaction of several proteins. For this reason, elucidation of ABA perception appeared to be very difficult and took considerable time and effort [110]. As depicted in Figure 2, ABA is sensed by its specific receptors named PYRABACTIN RESISTANCE/PYRABACTIN RESISTANCE-LIKE/REGULATORY COMPONENT OF ABA RECEPTORS (PYR/PYL/RCAR) [111,112]. In the absence of ABA, the receptors are inactive and their target proteins, the Ser/Thr phosphatases of the protein phosphatase 2C (PP2C) family, can block the activity of their downstream component, SUCROSE NONFERMENTING-1 RELATED PROTEIN KINASE 2 (SnRK2), thus preventing activity of the corresponding kinase [113,114], such as OST1 also known as SnRK2.6 [115]. In Arabidopsis, members of the PP2C family include ABI1/2, HAB1/2, HAI1/2/3, and AHG1/3 [116]. However, there is mounting evidence that other regulatory kinases could also be involved, such as the recently described RECEPTOR-LIKE PROTEIN KINASE 1 (RPK1) [117], and it would not be surprising if more pieces of the puzzle could be found in the future.

In the presence of ABA, the hormone binds to its receptors. This facilitates the binding of the receptors to PP2Cs, causing their inactivation. Inactivation of PP2C, in turn, leads to the release of SnRK2s from repression. SnRK2s activate themselves through their autophosphorylation loop, which renders them capable of phosphorylating their downstream target proteins, most notably transcription factors from the ABA-responsive element binding protein/ABRE-binding factor (AREB/ABF) families [118]. Furthermore, SnRKs phosphorylate plasma membrane proteins, including SLOW ANION CHANNEL 1 (SLAC1) or POTASSIUM CHANNEL IN ARABIDOPSIS THALIANA 1 (KAT1) [113,114]. Negative effectors modulate the intensity of the response and eventually terminate the signaling cascade to prevent an excessive positive feedback loop [116].

## 4. Plant Hormone Crosstalk

**Figure 3:**
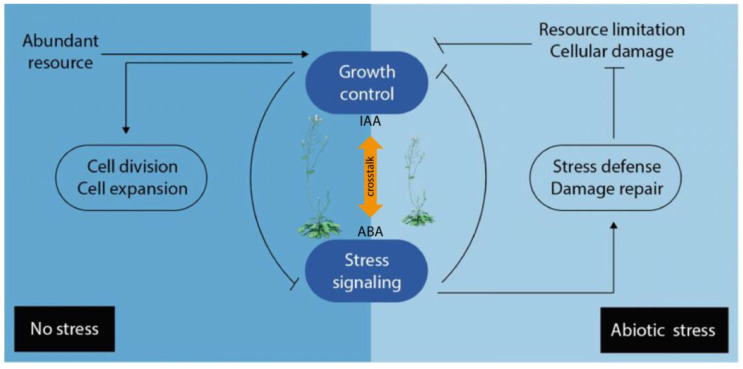
Simplified overview of the growth-defense trade-off under normal and abiotic stress conditions. When resources are abundant and stress is absent, growth signaling is activated, repressing stress signaling (**left** panel) in addition to promoting growth. Abiotic stress passively inhibits plant growth (**right** panel) by causing cellular damage and limiting resources (e.g., carbon dioxide, nutrients, and energy). Arrows indicate positive regulation, and bars indicate negative regulation. ABA: abscisic acid; IAA: indole-3-acetic acid.

Plant hormones do not act in entirely isolated manners, but in regulatory networks in which they operate in synergistic or antagonistic ways with each other. Consequently, the final condition of growth and development represents the net effect of the total hormone balance [119]. The term ‘hormone crosstalk’ describes how signal integration from multiple hormone inputs within a response network affects a common biological output [120]. Furthermore, it should be noted that the term ‘crosstalk’ refers to the interaction between two or more hormones or hormone pathways in cell signaling at the molecular level. However, hormone interplay would be an umbrella term that includes crosstalk too, and would encompass any hormonal interaction in the regulation of a physiological function, not necessarily antagonistic or synergistic [15]. Plant hormone crosstalk coordinates a sophisticated regulatory network, in a spatiotemporal fashion, to achieve specific physiological processes, regulate plant growth and development, as well as metabolism and defense [89]. Plant hormone networks are very complex and the change in one hormone, either through genetic- or metabolic changes, can cause alterations in others, whether in anabolism, catabolism, or sensitivity. The connections in these pathways through common elements are referred to as ‘nodes’ in the crosstalk [121]. These nodes are connected amongst each other and with other integrators that help to process internal or external signals, to steer adequate plant responses, and adjust plant growth and development to prevailing conditions. Different plant hormone signaling pathways can display redundancy, functional overlap, and multiple feedback loops combined with direct and indirect regulation between different routes. This complexity makes it extremely difficult to understand the complete outcome of a specific hormone signal [122]. It is well known that several phytohormones collaborate to adjust the developmental growth program to stress conditions, and hormone crosstalk is a novel approach that helps to understand these complex outcomes [123]. Phenotypic analyses of certain mutants suggest that hormones influence each other, both in synthesis and signaling. There have been different approaches to address and systematically understand hormone interactions, or crosstalk, forming complex networks that coordinate general plant growth and development [124]. Although our knowledge has been reviewed and substantially improved in the past decade, the ‘growth-defense trade-off’ phenomenon [125], by which plants allocate their limited resources either to development or defense against abiotic or biotic stresses remains a key concept in explaining how hormone levels are modulated to guarantee the survival of a plant [126]. For this reason, the exploration of the crosstalk between growth promoting and growth repressing plant hormones, such as auxin and ABA, appears to be particularly interesting (Figure 3). A deeper understanding of the intertwined biosynthesis of IAA and ABA will likely further our understanding of how plants prepare to withstand abiotic stresses, which biomolecules are involved in the process, and how they trigger the response to stress on the molecular level. The crosstalk between auxins and ABA might be fundamental in understanding the growth-defense trade-off phenomenon.

### 4.1. Crosstalk under Abiotic Stress Conditions

**Figure 4:**
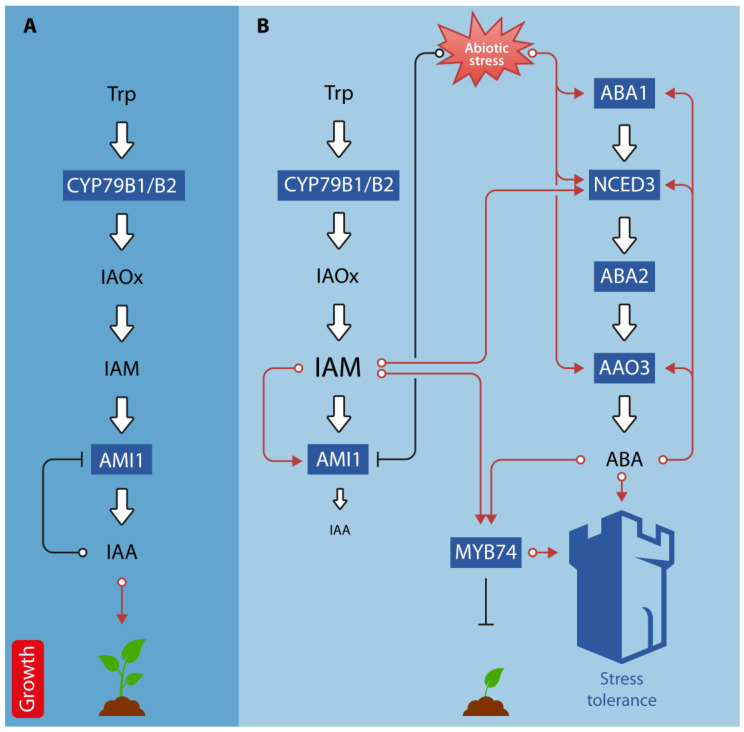
Schematic representation of auxin-ABA crosstalk under normal and abiotic stress conditions. (**A**) Under normal conditions, indole-3-acetamide (IAM) is converted to indole-3-acetic acid (IAA) by the virtue of AMI1, which promotes plant growth. (**B**) Under abiotic stress conditions, *AMI1* expression is repressed, which translates into a reduced IAA biosynthesis rate and the elevation of cellular IAM levels. IAM, in turn, is inducing ABA biosynthesis through the transcriptional activation of *NCED3*. The expression of several ABA biosynthesis-related genes, including *NCED3*, *ABA1*, and *AAO3*, is also directly stimulated by abiotic stress stimuli. Both, IAM and ABA, trigger the expression of the negative growth regulator MYB74. Increased ABA and MYB74 levels promote the abiotic stress tolerance of the plant. Red lines indicate stimulation of gene expression/growth, while black lines refer to the repression of gene expression and plant growth, respectively. The white bold arrows pointing to IAA and ABA, respectively, refer to the biosynthetic pathways of both hormones. Metabolites are given in black letters (letter size of IAM and IAA shows changes in endogenous content). Involved genes are given in dark blue boxes. The figure also shows positive feed-back loops for the regulation of ABA biosynthesis genes and highlights the transcriptional regulation of AMI1 by its substrate, IAM, and reaction product, IAA.

The interactions between auxin and ABA regulate a wide variety of developmental processes, such as seed germination, cell expansion, hypocotyl elongation, root elongation, lateral root formation, and cotyledon growth [127]. In addition, there is compelling evidence for the involvement of auxin-ABA crosstalk in the regulation of abiotic stress-related responses. For example, auxin homeostasis influences ABA biosynthesis and associated drought stress responses in rice [128,129]. In white clover, exogenous IAA treatment significantly increases ABA contents, upregulate the expression of drought stress responsive genes, including *DEHYDRATION-RESPONSIVE ELEMENT BINDING 2* (*DREB2*), the transcription factor genes *bZIP11*, *MYB14*, *MYB48*, *WRKY2*, *WRKY56*, and *RESPONSIVE TO DESICCATION* 22 (*RD22*), along with some auxin responsive genes, such as the *Gretchen Hagen 3* genes *GH3.1* and *GH3.9*, as well as the Aux/IAA gene *IAA8*. At the same time, IAA treatment triggered the downregulation of leaf senescence-associated genes, such as *SENESCENCE-ASSOCIATED GENE 101* (*SAG101*) and *SAG102* [130]. Small auxin upregulated RNAs (SAURs) are recognized as auxin-responsive genes involved in the regulation of abiotic stress adaptive growth. In Arabidopsis, *SAUR32* was dominantly expressed in roots and was highly induced by ABA and drought [131]. *SAUR32* interacts with the clade-A PP2C proteins HAI1 and AIP1 to regulate ABA sensitivity. A barley mutant, *nec1*, that is defective in an *HLM* gene that encodes the CYCLIC NUCLEOTIDE-GATED ION CHANNEL 4, has been reported to over accumulate IAA and exhibit changes in stomatal regulation in response to exogenous auxin. The Arabidopsis orthologous mutant *dnd2* shows increased salt tolerance, over-accumulation of both IAA and ABA, and displays related phenotypic and physiological changes, such as reduced stomata size, higher stomatal density, and stomatal index [132]. Interestingly, a recent study reported that the increase in IAM content in the *ami1* mutant leads to an induction of *NCED3* expression and, consequently, to significantly elevated levels of ABA [59]. Furthermore, *ami1* mutants were shown to have altered osmotic stress responses. Thus, it must be concluded that IAM is an additional hub that links the biosynthesis pathways of IAA and ABA and, thus, contributes to the crosstalk between these two plant hormones. Furthermore, Ortiz-García et al. [133] provided evidence for the co-regulation of the transcription factor *MYB74*, which is reported to be involved in osmotic stress responses, by IAM and ABA (Figure 4).

### 4.2. Further Examples for Auxin-ABA Crosstalk

The extensive interaction of ABA with other hormones in the regulation of stress responses, growth, and development is well documented [134]. Nonetheless, there are still large gaps in our knowledge on the crosstalk of ABA with other phytohormones, and sometimes we lack consensus on a given interaction. Crosstalk between two hormones can exist in a given process but not in another. For example, ABA and ethylene are antagonizing hormones at the germination level, but appear to interact in controlling stomatal closure [135]. In fact, ABA and ethylene have been commonly understood as antagonists, but more recent studies suggest that they could operate in parallel or even interact synergistically [89]. In addition, multiple and seemingly unrelated stimuli can affect the same hormonal pathways, e.g., many sugar germination assays revealed loci allelic to ABA, since they had been already described as ABA-responsive genes, but not all ABA-responsive genes are sugar-responsive [136]. Interactions between ABA and auxins are recognized to control essential plant programs, including seed germination [137] and seedling establishment [138], stomata closure [139], as well as meristematic activity of the main root and lateral root development [140]. Germination is a terminal process, irreversible in nature, and therefore influenced by countless inputs, both exogenous and endogenous [124] and must begin in a favorable environment. Although this field of research is still in its infancy, some nodes have been uncovered. In most of the examples of auxin-ABA crosstalk described so far, ABA seems to act upstream of auxin [127], but there seem to be hints that auxin-ABA crosstalk is more mutual and intertangled, and in some instances the master regulation of ABA has not been unequivocally proven yet. As a classical example, in Arabidopsis, ABA acts upstream of ARF2 which, in turn, directly regulates the expression of the homeodomain gene *HOMEOBOX PROTEIN 33* (*HB33*) in both seed germination and primary root growth [141]. In fact, one of the well-investigated mechanisms in which ABA influences auxin signaling is through the regulation of ARFs, including post-transcriptional modulation [142]. For example, ABA induces ARF6 ubiquitination, leading to protein degradation [143]. ABI3 would constitute an interesting node, because this particular protein is essential for multiple gene networks that control developmental processes [144,145] and is considered the major regulator of seed dormancy in plants. In Arabidopsis, auxin is known to control dormancy through stimulation of ABA signaling by inducing *ABI3* expression [146]. Auxin acts upstream of *ABI3* by recruiting auxin response factors ARF10 and ARF16. In turn, ARF10 is negatively regulated by *miRNA160* [147]. ARF10/16 promote *ABI3* transcription, whose transcripts are high in dormant seeds and lowered after germination, regulating the seed dormancy in synergy with ABA [146], but not by direct binding, raising the question of which intermediate transcription factors operate in this pathway. Closing the feedback loop, ABI3 negatively regulates *MIR160B*, amongst other miRNA genes [148]. Furthermore, auxin-induced *ABI3* expression has been reported to be required for the formation of somatic embryogenesis, because it induces the expression of embryo identity genes through, at least, ARF10/16 activation [148]. Regarding its connection with ARFs, *ABI3* has also been described to be negatively regulated by the auxin signaling repressor IAA8, whose accumulation promotes seed germination [149]. The authors suggest that the binding of IAA8 to the *ABI3* promoter through ARFs suppresses *ABI3* transcription during seed germination, but the exact ARF proteins have not yet been identified. Interestingly, IAA8 has been shown to interact with ARF6 and ARF8 in modulating JA levels during flower development [150]. In another example, ABI5 is not only a known hub for ABA-mediated abiotic stress responses and crosstalk with GAs, BRs, and JAs [151], but also an important node connecting ABA and sugar sensing through the TARGET OF RAPAMYCIN (TOR) kinase, which is activated by auxins [152]. In fact, the reciprocal regulation of the TOR kinase and ABA receptors has been proposed to balance plant growth and stress responses [153] and inhibition of TOR alters auxin signaling [154]. For example, at high concentrations of glucose, ABI5 suppresses the accumulation of PIN1 in the root meristem, thus decreasing auxin activity and inhibiting root elongation [155]. ABA also suppresses auxin-mediated primary root elongation under abiotic stress through upregulation of *ABI5*, which acts by inducing degradation of PIN2 proteins [156]. Complementary, salt stress significantly decreases the expression of the *PIN1*, *PIN3*, and *PIN7* genes and promotes the stabilization of AUXIN RESISTANT 3 (AXR3)/IAA17 through nitric oxide (NO) [157]. However, although ABI5 is known to be regulated by NO at both the transcriptional and protein levels [158,159] the precise involvement of ABA in this pathway remains unknown. Given its utmost importance, *ABI5* expression and the biological half-life of the protein are tightly regulated [151], and other transcription factors fine-tune its transcription, such as the rice APETALA2-type transcription factor SALT AND ABA RESPONSE ERF 1 (OsSAE1), which acts as a positive regulator of seed germination and salt tolerance in rice by suppressing the expression of *OsABI5* [160]. In another example, mediator (MED) proteins, such as MED25, positively contribute to auxin signaling, but interact with ABI5 to negatively regulate ABA responses [161,162], while MED16 competes with MED25 in physical interactions with ABI5 and is a positive regulator of ABA responses [163]. ABI4 would serve as another connection point, being stabilized by stress, ABA, and phosphorylation [164], and is also known to receive auxin inputs [165], at least in roots, where its expression is induced by ABA and cytokinin and repressed by auxin [166], but its role in crosstalk needs further exploration [167]. Interestingly, PIN1 levels are reduced under abiotic stress in an ABA-dependent manner, e.g., after mannitol treatment [168], since ABI4 mediates ABA- and the cytokinin-mediated inhibition of lateral root formation by reducing the expression of *PIN1*, which plays a role in polar auxin transport necessary for root development [166], even over-riding other hormonal signals, such as ethylene [169]. In turn, *ABI4* is also regulated, for example, by WRKY46, which integrates ABA-dependent and ABA-independent abiotic stress responses and, thus, controls auxin-related gene expression [170]. ABI4 may also be key to integrating ABA, auxin, and reactive oxygen species (ROS) signaling via ASCORBATE PEROXIDASE 6 (APX6) in seeds [171,172] and controls auxin levels through upstream involvement of YUC4 [173], although the precise mechanisms are not yet clear. In rice, the *OsPIN2* mutant *wavy root 1* (*war1*) is defective in auxin transport and auxin distribution at root tips, leading to loss of gravitropic perception, but also shows increased sensitivity to ABA in seed germination, increased ABA levels, and changes in ABA-associated gene expression in roots [174]. A subsequent study later revealed that ABA reduces the amount of PIN2 in the membrane by suppressing the expression of *PIN2* rather than accelerating the degradation of PIN2 [140]. In rice as well, *Os*IAA20 is positively regulated by abiotic stress conditions and exogenous ABA treatment, and contributes positively to abiotic stress tolerance: reduces water loss, improves seed germination, decreases the Na^+^/K^+^ ratio, increases the proportion of closed stomata, and also enhances growth in different developmental stages [175]. Remarkably, *Os*IAA18 also confers salt and drought tolerance, at least by increasing proline biosynthesis and reducing ROS accumulation, through an ABA-dependent pathway, therefore acting upstream of ABA-signaling pathways [176], even the heterologous expression of the rice gene in Arabidopsis [177], and heterologous expression of the grapevine *VvIAA18* in tobacco enhances drought tolerance, as measured by upregulation of salt stress-responsive genes, including *PYRROLINE-5-CARBOXYLATE SYNTHASE* (*P5CS*), *LATE EMBRYOGENESIS ABUNDANT PROTEIN 5* (*LEA5*), *SUPEROXIDE DISMUTASE* (*SOD*) and *PEROXIDASE* (*POD*) under drought stress, as well as higher SOD and POD activities [178]. However, whether these Aux/IAAs are directly activated by ABA has not been elucidated yet. The regulatory model in which salt/drought stress regulates Aux/IAAs exists also in Arabidopsis, where under drought conditions, DREB2A and DREB2B directly regulate the expression of the Aux/IAA genes *IAA5*, *IAA6*, and *IAA19* [179].

## 5. Open Challenges in Auxin-ABA Crosstalk

Despite recent discoveries that shed light on the intricacies of auxin-ABA crosstalk, there are remaining knowledge gaps that need to be closed. For example, the role of indeterminate domain (IDD) transcription factors in abiotic stress responses have recently been discovered. IDD14 can physically interact with ABF1-4, promoting their transcriptional activities by forming a protein complex that positively regulates drought-stress responses [180]. Numerous rice IDDs can also respond to auxin and ABA [181]. Three Arabidopsis IDD transcription factors, IDD14, IDD15, and IDD16, are known to cooperatively regulate spatial auxin accumulation by directly targeting *YUC5*, *TAA1*, and *PIN1* to promote auxin biosynthesis and transport [182], but it is not clear how this could influence auxin-ABA crosstalk. Another very recent study demonstrated that the ABA signaling-related transcription factor bZIP46 directly controls the expression levels of *YUC8* in rice, and genetic studies provided additional evidence that ABA uses auxin as a downstream signal to modify root elongation and radial expansion [183]. One further work highlighted that the transcription factor WRKY41 is an important regulator of *ABI3* expression during seed dormancy, acting not downstream of ABA signaling, but probably in parallel [184]. However, the stimuli that induce *WRKY41* are not yet clear, and their elucidation might reveal new interactions. *PIN3* expression is also directly inhibited by HAT2, an HD-Zip II transcription factor, whose expression is upregulated by osmotic stress, at least in roots, thus inhibiting meristem growth [185], and its expression is also downregulated in response to NaCl [186], but the exact mechanism remains uncertain. Auxin-ABA interactions remain particularly understudied in relation to cold responses, which are mediated by ABA [187]. Low temperatures inhibit root growth, due to the fact that endosomal trafficking of auxin efflux carriers is inhibited [188], and partially by repressing the expression of *PIN1/3/7* and auxin biosynthesis, a possible crosstalk node would be the ARABIDOPSIS RESPONSE REGULATOR 1 (ARR1) transcription factor [189], in which auxin, cytokinin, and ABA signaling seem to converge [190], but the mechanisms remain elusive [191].

## Data Availability

Data sharing is not applicable. No new data were generated or analyzed in this study.
